# Normal ranges of right atrial strain by contemporary echocardiography software: a prospective comparative cohort study

**DOI:** 10.1007/s12574-025-00689-9

**Published:** 2025-04-23

**Authors:** Ankit Agrawal, Aro Daniela Arockiam, Elio Haroun, Tiffany Dong, Joseph El Dahdah, Muhammad Majid, Mohammad Alamer, Sharmeen Sorathia, Richard A. Grimm, Patrick Collier, Leonardo L. Rodriguez, Zoran B. Popovic, Brian P. Griffin, Tom Kai Ming Wang

**Affiliations:** Section of Cardiovascular Imaging, Department of Cardiovascular Medicine, Sydell and Arnold Miller Heart, Vascular, and Thoracic Institute, Cleveland Clinic, 9500 Euclid Avenue, Main Campus, J1-5, Cleveland, OH 44195 USA

**Keywords:** Strain, Right atrium, Echocardiography, Right atrial strain

## Abstract

**Background:**

Right atrial (RA) strains (RASr, RAScd, and RASct) are increasingly used in clinical and research settings, such as heart failure and pulmonary hypertension, but their feasibility and reference ranges across different strain software vendors are not well established. We aim to evaluate and compare two-dimensional RA strain values, reference ranges, and related factors across four strain software vendors in healthy subjects.

**Methods:**

Healthy subjects (*n* = 100) undergoing echocardiography during January–April 2023 were prospectively studied, with equal numbers by age groups, gender, and GE versus Philips scans. RA strains were quantified using TomTec version 51.02 (Autostrain LA), EchoPAC version 206 (AFI-LA), Velocity-Vector Imaging (VVI) version 2.00, and Epsilon software (5.0.2.11295) for statistical analyses.

**Results:**

Overall means and lower limits of normal (LLNs) of each type of RA strain by strain vendor, age group, sex, and scanner vendor were reported. For example, RASr (%) means and LLNs (95% confidence intervals) were 41.2 (38.5, 43.0) and 29.6 (26.5, 32.7) for TomTec, 35.9 (34.4, 37.3) and 27.0 (24.5, 29.5) for EchoPAC, 44.8 (42.3, 47.3) and 27.6 (23.3, 31.9) for VVI, and 38.9 (36.7, 41.0) and 25.5 (21.7, 29.3) for Epsilon, respectively. Linear mixed model regression showed EchoPAC and VVI had significantly lower RASr and higher RAScd magnitude than TomTec, with older age linked to lower RASr and RAScd magnitudes.

**Conclusion:**

TomTec and VVI were vendor-neutral for measuring RA strains, while EchoPAC worked only on GE scans. Normal values, lower limits of normal, and related factors for RA strain measurements by vendor were established for clinical use.

## Introduction

Speckle-tracking echocardiography has evolved into a pivotal component of routine transthoracic echocardiography (TTE) for evaluating chamber function using strain [1]. Right atrial (RA) strains, including right atrial reservoir (RASr), conduit (RAScd), and contractile (RASct) strains, are increasingly utilized biomarkers for RA function, with evolving clinical applications and prognostic utility in several cardiovascular conditions, such as heart failure, pulmonary hypertension (PH), or cardiac amyloidosis [2–5]. Similarly, left-ventricular, left atrial, and right-ventricular strains have demonstrated clinical utilities in various cardiovascular diseases, offering insights into disease progression, prognosis, and treatment response for heart failure with reduced and preserved ejection fraction, atrial fibrillation, coronary heart disease, valvular heart disease, cardio-oncology, hypertension, and PH [6, 7]. A few meta-analyses have also been published but with different lower limits of normal (LLN), meaning the normal range of RA strain measurements remains poorly established, including for each strain software, and factors affecting RA strain measurements in healthy subjects are poorly understood [8, 9]. An additional noteworthy limitation of strain measurement in clinical practice was the historical dependence on strain software tied to specific vendors. This restriction meant that such software could only be utilized with TTE machines from those vendors. In contrast, vendor-neutral software solutions, like Velocity-Vector Imaging (VVI) and Epsilon, were primarily confined to research settings, limiting their widespread clinical application. However, recent advancements in clinical strain software have aimed to address these shortcomings, although external evaluation of these properties is lacking.

The primary objective of this prospective study is to measure RA strains in a healthy subject population using four contemporary strain software programs to determine means and LLNs and biological parameters that influence RA strain values.

## Materials and methods

### Study population

From January to April 2023, we conducted a prospective evaluation of 100 healthy subjects aged over 18 years who underwent clinically indicated and complete TTE at the Cleveland Clinic. The inclusion criteria comprised 50 male and 50 female subjects, with 20 subjects in each of the following age groups: 18–29, 30–39, 40–49, 50–59, and 60 + years. Additionally, 50 subjects were scanned using GE Vivid 7 or E9 (GE Medical, Milwaukee, WI, USA) echocardiography machines, and the other 50 with Philips EPIQ 7C (Philips Medical Systems, Bothell, WA, USA) echocardiography machines. This distribution ensured an equal number of subjects for each category based on sex, age group, and scanner. To be included in the study, subjects needed to have adequate atrial views from the apical 4-chamber, as assessed by two independent echocardiography readers. Individuals with any history of cardiovascular diseases (including coronary heart disease, heart failure/cardiomyopathy, valvular heart disease of at least moderate severity, arrhythmia, congenital heart disease, pericardial diseases, cardiac medications, surgery, interventions, and devices), hypertension, diabetes, stroke, PH, chronic lung, kidney, liver, vascular, and inflammatory diseases, malignancies, chemotherapy, and radiation therapy were excluded from the study. We recorded relevant clinical characteristics of the healthy subjects who presented for screening examinations. The study received approval from our Institutional Review Board (IRB 23–207), and subject consent was waived. Subjects and the public were not directly involved in this study’s design, conduct, or reporting. The study primarily focuses on the technical evaluation of RA strain measurements in a healthy subject population, intending to establish normative values and understand biological influences.

### Echocardiography

TTE scans were conducted using either GE Vivid E95 (GE Medical, Milwaukee, WI, USA) or Philips EPIQ-CVX version 9.0 (Philips Medical Systems, Bothell, WA, USA) scanners. For RA evaluation, the apical 4-chamber view was employed from the complete TTE study, ensuring that the RA was not foreshortened and that endocardial definition was visible throughout the entire cardiac cycle. RA strain was analyzed for all subjects using strain vendors TomTec version 51.02 (Autostrain LA), EchoPAC version 206 (AFI), VVI software, and Epsilon (5.0.2.11295) to calculate RA strain. RASr represents the atrium’s ability to store blood during ventricular systole. RAScd reflects the passive emptying of the atrium during early ventricular diastole. RASct measures the active contraction of the atrium during late ventricular diastole. To assess intra-reader variability, a set of 20 randomly chosen subjects had their RA strains measured on all three strain vendors by the same reader. Inter-reader variability was assessed by other readers independently measuring the RA strains of the same set of subjects.

### Statistical analysis

Baseline continuous variables were reported using mean ± standard deviation, while categorical variables were presented as frequency (percentage). RA strain means and LLNs with their 95% confidence intervals were calculated for the entire cohort by each strain vendor software, and mean ± standard error was calculated for each age group, sex, and TTE scanner vendor category. The LLN for RA strains was determined as the 95th percentile strain value in the 100-subject cohort at the lower end of the strain value magnitude, for instance, less negative for RA strain. The standard error for the LLN was computed using the formula SE LLN = √(SDmean^2 × (1/n + 2/(n-1))/12. Intra- and inter-reader variability was calculated on 20 subjects using the standard error of measurement (SEM) method for each type of RA strain and strain software. Linear mixed model multivariable regression analyses were performed to identify clinical and echocardiographic factors associated with RA strain measurements by different strain vendor software, reporting beta-coefficients and 95% confidence intervals (CI). Statistical analyses and graphing were conducted using SPSS (version 24, IBM, Chicago, IL, USA) and Prism (version 8, GraphPad, San Diego, CA, USA) software. A *P* value < 0.05 was considered statistically significant, and all tests were two-tailed.

## Results

Baseline clinical and echocardiographic characteristics are listed in Table 1. The mean age was 45 ± 15 years, distributed evenly between males and females. Most of our population were mostly white subjects (83%), while African Americans, Asians, and other races constituted 5, 6, and 6%, respectively. The participants exhibited a higher-than-normal body mass index (BMI), averaging 26 ± 5 kg/m^2^, and a corresponding body surface area (BSA) of 1.92 ± 0.2. The mean heart rate, systolic, and diastolic blood pressure were 70 ± 13 beats/min,122 ± 17 mm Hg and 74 ± 10 mm Hg, respectively. The mean indexed left-ventricular end-diastolic and left-ventricular end-systolic volumes were 50 ± 11 and 19 ± 6, respectively. The mean indexed left-ventricular stroke volume indexed stroke volume, left-ventricular ejection fraction, and left-ventricular mass indexed was 31 ± 7, 62 ± 5, and 65 ± 16, respectively. Additionally, the mean speckle-tracking echocardiography frame rate was 52 ± 10 Hz. The central illustration displays the different strain software and a reference for RA strain.

**Table 1:** Cohort characteristics

Number of subjects	100
Clinical	
Age (years)	45 ± 15
Female (%)	50 (50%)
Ethnicity	
White	83 (83%)
Black	5 (5%)
Asian	6 (6%)
Other	6 (6%)
Weight (kg)	79 ± 20
Height (m)	1.71 ± 0.15
Body mass index (kg/m^2^)	26 ± 5
Body surface area (m^2^)	1.92 ± 0.26
Heart rate (beats/minute)	70 ± 13
Systolic blood pressure (mmHg)	122 ± 17
Diastolic blood pressure (mmHg)	74 ± 10
Creatinine (mg/dL)	0.87 ± 0.20
Echocardiography	
Frame rate (Hertz)	52 ± 10
Left-ventricular end-diastolic volume indexed (mL/m2)	50 ± 11
Left-ventricular end-diastolic volume indexed (mL/m2)	19 ± 6
Left-ventricular stroke volume indexed (mL/m2)	31 ± 7
Left-ventricular ejection fraction (%)	62 ± 5
Right-ventricular end-diastolic basal diameter (cm)	3.4 ± 0.7
Tricuspid annular plane systolic excursion (cm)	2.2 ± 0.4
Right-ventricular lateral annular tissue Doppler S´ (cm/s)	13.1 ± 2.3
Right-ventricular systolic pressure (mm Hg, *n* = 38, remaining unable to measure)	22 ± 5
Left atrial volume index (mL/m^2^)	24 ± 7
Right atrial volume index (mL/m^2^)	18 ± 7

Mean ± standard deviation or frequency (percentage)

Table 2 presents the means and LLN for RA strain, as determined by strain software in echocardiography. Means (95%CIs) and LLNs for RASr were 41.2% (38.5%, 43.0%) and 29.6% (26.5%, 32.7%) for TomTec, 35.9% (34.4%, 37.3%) and 27.0% (24.5%, 29.5%) for EchoPAC, 44.8% (42.3%, 47.3%) and 27.6% (23.3%, 31.9%) for VVI, and 38.9% (36.7%, 41.0%) for Epsilon. Intra- and inter-reader variability were 2.19–9.67% and 4.15–11.54% for RASr.

**Table 2:** Right atrial strain means and lower limits of normal and 95% confidence intervals by echocardiography strain software in healthy subjects

Strain vendor software	TomTec	EchoPAC	VVI	Epsilon
Number of subjects	100	50 (GE scans only)	100	100
RASr (%)				
Mean	41.2 (38.5, 43.0)	35.9 (34.4, 37.3)	44.8 (42.3, 47.3)	38.9 (36.7, 41.0)
LLN normal	29.6 (26.5, 32.7)	27.0 (24.5, 29.5)	27.6 (23.3, 31.9)	25.5 (21.7, 29.3)
Intra-reader variability	4.9	2.1	9.6	4.1
Inter-reader variability	7.4	4.1	10.2	7.0
RAScd (%)				
Mean	−25.9 (−27.5, −24.4)	−22.9 (−24.2, −21.5)	−27.6 (−29.8, −25.4)	−24.5 (−26.4, −22.7)
LLN normal	−14.3 (−17.1, −11.5)	−14.0 (−16.4 −11.6)	−13.2 (-17.0, -9.4)	−12.5 (−15.7, −9.3)
Intra-reader variability	5.1	2.3	11.1	4.1
Inter-reader variability	7.7	3.8	7.8	7.0
RASct (%)				
Mean	−15.3 (−16.2, −14.4)	−12.4 (−13.5, −11.3)	−17.1 (−18.6, −15.5)	−14.3 (−15.2, −13.5)
LLN normal	−10.2 (−11.7, −8.7)	−8.0 (−9.9, −6.1)	−8.0 (−10.7, −5.3)	−9.6 (−11.1, −8.1)
Intra-reader variability	3.5	1.6	5.8	3.0
Inter-reader variability	3.2	5.4	7.1	7.6

The mean or lower limits of normal (LLN) and their 95% confidence intervals (95% CI) are presented. Intra- and inter-reader variability is assessed using the standard error of measurement. *RASr* right atrial reservoir strain, *RAScd* right atrial conduit strain, *RASct* right atrial contractile strain, and *VVI* velocity vector imaging

**Fig. 1:**
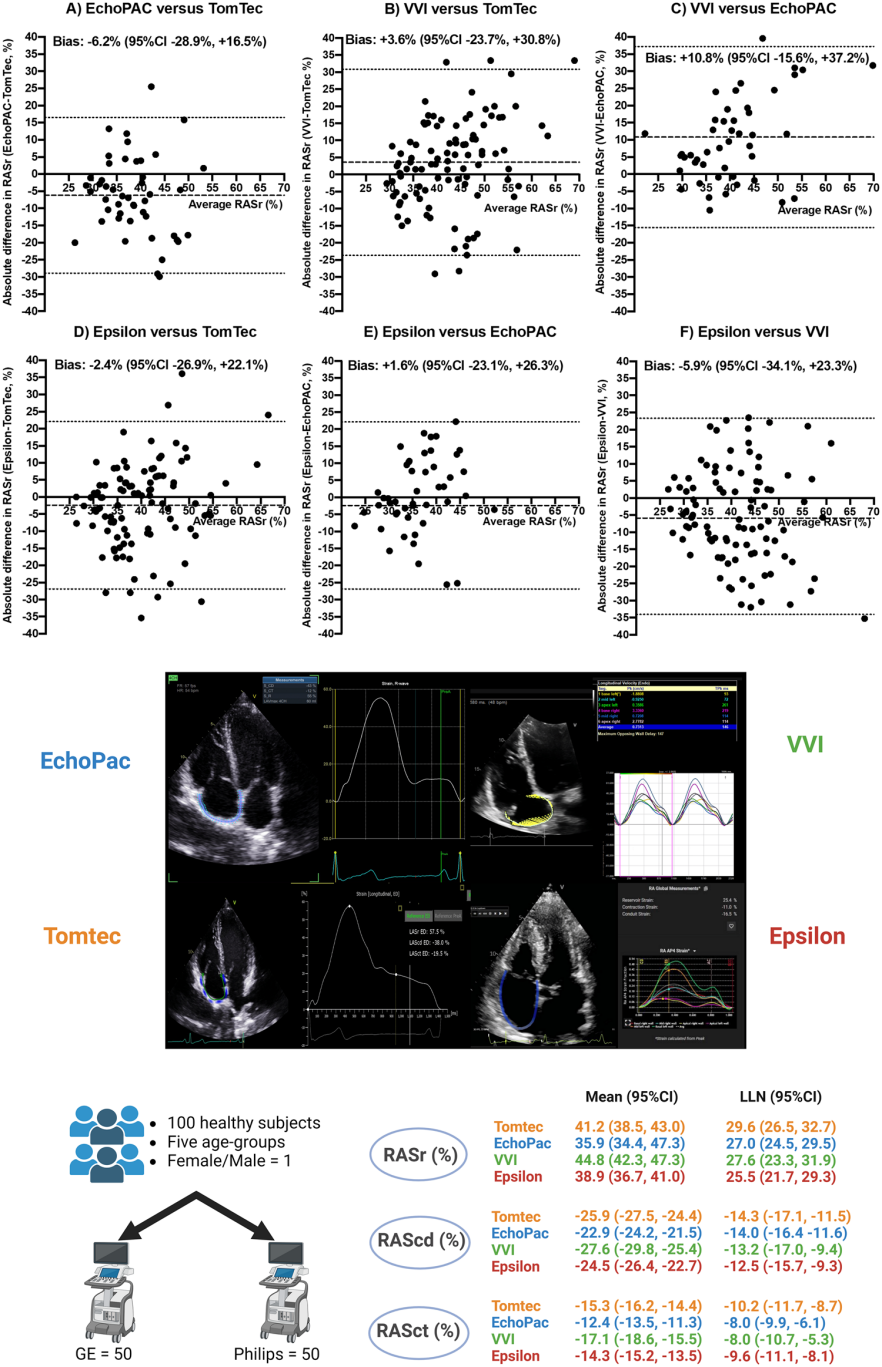
Bland–Altman plots of pairwise vendor software (TomTec, EchoPAC, VVI, and Epsilon) comparisons of RASr on TTE in healthy subjects. *Central illustration*: Central figure depicting the summary of our findings

RA strains by age group, sex, and scanner vendor subgroups are reported in Table 3. RASr and RAScd tended to decrease in magnitude as age increased. RASr and RAScd also tended to be higher in magnitude for women than men, except for EchoPAC. No significant trends were observed for RASct and for scanner type. Pairwise comparisons of RA strains between strain vendors are illustrated by Bland–Altman plots in Fig. 1.

**Table 3:** Right atrial strain means measured by different echocardiography strain software in age, sex, and echocardiography scanner vendor subgroups in healthy subjects

Strain vendor software	TomTec	EchoPAC	VVI	Epsilon
Number of subjects	100	50 (GE scans only)	100	100
RASr (%)				
Overall (*n* = 100)	41.2 ± 0.9	35.9 ± 0.7	44.8 ± 1.3	38.9 ± 1.1
Age group (years)				
18–29 (*n* = 20)	44.4 ± 1.9	36.1 ± 3.9	51.4 ± 3.8	43.6 ± 1.9
30–39 (*n* = 20)	38.6 ± 2.0	38.2 ± 3.0	45.3 ± 2.4	37.0 ± 2.9
40–49 (*n* = 20)	43.1 ± 1.6	35.3 ± 1.7	42.4 ± 2.8	38.4 ± 3.1
50–59 (*n* = 20)	43.0 ± 2.4	33.0 ± 1.3	41.7 ± 2.2	37.9 ± 2.3
60 + (*n* = 20)	37.2 ± 1.8	32.3 ± 1.6	43.1 ± 2.3	37.4 ± 1.7
Sex				
Male (*n* = 50)	41.0 ± 1.3	33.0 ± 2.0	43.8 ± 1.7	36.9 ± 1.6
Female (*n* = 50)	41.5 ± 1.2	37.3 ± 1.5	45.7 ± 1.9	40.8 ± 1.5
Scanner				
GE (*n* = 50)	41.4 ± 1.2	35.1 ± 1.3	46.3 ± 1.9	36.6 ± 1.3
Philips (*n* = 50)	41.1 ± 1.3	Non-measurable	43.3 ± 1.7	41.2 ± 1.8
RAScd (%)				
Overall (*n* = 100)	−25.9 ± 0.8	−22.9 ± 0.7	−27.6 ± 1.1	−24.5 ± 1.0
Age group (years)				
18–29 (*n* = 20)	−30.2 ± 1.9	−27.4 ± 2.9	−36.3 ± 3.3	−29.2 ± 1.9
30–39 (*n* = 20)	−24.3 ± 1.8	−26.0 ± 2.3	−24.9 ± 1.8	−24.3 ± 2.6
40–49 (*n* = 20)	−27.8 ± 1.7	−21.8 ± 1.6−	−28.8 ± 2.4	−25.4 ± 2.8
50–59 (*n* = 20)	−25.5 ± 1.9	−18.4 ± 1.4	−24.0 ± 1.9	−23.4 ± 1.9
60 + (*n* = 20)	−21.9 ± 1.4	−19.9 ± 2.0	−24.0 ± 1.6	−20.1 ± 1.6
Sex				
Male (*n* = 50)	−25.9 ± 1.2	−20.6 ± 1.5	−26.1 ± 1.4	−23.1 ± 1.5
Female (*n* = 50)	−26.0 ± 1.1	−25.1 ± 1.3	−29.1 ± 1.7	−25.8 ± 1.3
Scanner				
GE (*n* = 50)	−26.1 ± 1.2	−22.8 ± 1.0	−28.7 ± 1.8	−22.3 ± 1.1
Philips (*n* = 50)	−25.8 ± 1.1	Non-measurable	−26.6 ± 1.3	−26.7 ± 1.6
RASct (%)				
Overall (*n* = 100)	−15.3 ± 0.5	−12.4 ± 0.5	−17.1 ± 0.8	−14.3 ± 0.4
Age group (years)				
18–29 (*n* = 20)	−14.2 ± 1.0	−13.6 ± 1.3	−14.4 ± 1.8	−14.5 ± 3.9
30–39 (*n* = 20)	−14.2 ± 0.8	−12.0 ± 1.2	−20.4 ± 1.9	−12.7 ± 1.0
40–49 (*n* = 20)	−15.3 ± 0.8	−13.6 ± 1.0	−13.6 ± 0.9	−13.3 ± 1.0
50–59 (*n* = 20)	−17.5 ± 1.3	−10.3 ± 3.0	−17.8 ± 2.1	−14.5 ± 0.9
60 + (*n* = 20)	−15.3 ± 1.0	−12.2 ± 0.9	−19.1 ± 1.7	−16.9 ± 0.9
Sex				
Male (*n* = 50)	−15.1 ± 0.6	−13.9 ± 0.7	−17.4 ± 1.3	−13.7 ± 0.6
Female (*n* = 50)	−15.5 ± 0.6	−10.9 ± 1.4	−16.7 ± 0.9	−15.0 ± 0.7
Scanner				
GE (*n* = 50)	−15.3 ± 0.6	−12.4 ± 0.8	−17.4 ± 1.0	−14.2 ± 0.6
Philips (*n* = 50)	−15.3 ± 0.7	Non-measurable	−16.7 ± 1.2	−14.5 ± 0.7

Means ± standard errors. *RASr* right atrial reservoir strain, *RAScd* right atrial conduit strain, *RASct* right atrial contractile strain, *VVI* velocity vector imaging

Table 4 presents the results of the linear mixed model regression analyses for clinical and echocardiography parameters associated with RAS measurements. Older age per 10 years was associated with lower RASr with β (95%CI) of −1.43 [−2.40, −0.46]) and higher RAScd with β (95% CI) of 1.91 (1.09, 2.73). EchoPAC had a significantly lower magnitude of RASr and RASct than TomTec, with β (95% CI) of -6.29 (-9.99, -2.59) and 2.91 (0.87, 4.95), respectively. VVI had a significantly higher magnitude of RASr and RASct than TomTec with β (95%CI) of 3.55 (0.74, 6.36) and −1.76 (−3.30, −0.22), respectively, while Epsilon and TomTech were similar. Sex, body mass index, heart rate, frame rate, scanner vendor, and right atrial volume index were not significantly associated with RA strain measurements.

**Table 4:** Linear mixed model multivariable regression longitudinal analyses of clinical and echocardiography factors associated with right atrial reservoir, conduit, and contractile strain in healthy subjects

Covariate\model	RASr	RAScd	RASct
Age (per 10 years)	**−1.43 (−2.40, −0.46)**	**1.91 (1.09, 2.73)**	−0.34 (−0.82, 0.01)
Female	0.55 (−2.44, 3.54)	−0.69 (−3.23, 1.85)	0.73 (−0.79, 2.25)
Body mass index (per 10 kg/m^2^)	−0.29 (−2.97, 2.39)	0.66 (−1.66, 2.98)	−0.09 (−1.41, 1.23)
Heart rate (per 10 beats/minute)	−0.28 (−1.46, 0.90)	0.22 (−0.78, 1.21)	−0.08 (−0.66, 0.51)
Systolic blood pressure (per 10 mmHg)	0.59 (−0.29, 1.48)	−0.23 (−0.95, 0.50)	**−0.52 (−0.98, −0.01)**
Frame rate (per 10 Hz)	−0.46 (−1.78, 0.87)	0.24 (−0.89, 1.37)	−0.06 (−072, 0.61)
Right atrial volume index (per 10 mL/m^2^)	−0.17 (−2.15, 2.11)	−0.60 (−2.44, 1.25)	0.32 (−0.78, 1.42)
Philips scanner (versus GE)	0.68 (−2.19, 3.54)	−0.80 (−3.20, 1.61)	0.04 (−1.40, 1.47)
EchoPAC (versus TomTec) strain software	**−6.29 (−9.99, −2.59)**	2.97 (−0.15, 6.09)	**2.91 (0.87, 4.95)**
VVI (versus TomTec) strain software	**3.55 (0.74, 6.36)**	−1.65 (−4.01, 0.68)	**−1.76 (−3.30, −0.22)**
Epsilon (versus TomTec) strain software	−2.38 (−5.19, 0.43)	1.43 (−0.92, 3.78)	0.95 (−0.58, 2.49)

Beta-coefficients (95% confidence interval). *P* value < 0.05 in bold. *RASr* right atrial reservoir strain, *RAScd* right atrial conduit strain, *RASct* right atrial contractile strain, *VVI* velocity vector imaging

## Discussion

This prospective study yielded several important findings. First, TomTec, VVI, and Epsilon were able to measure RA strain on both GE and Philips scans; however, EchoPAC could only measure RA strain on GE but not Philips scans. Means and LLNs with 95% CIs for each type of RA strain by strain vendor were reported to determine abnormal from normal strain. Mean RA strains by age groups, sex, and scanner vendor were also reported. Finally, key factors, especially age and strain vendor, were found to significantly affect RA strain measurements, which have important clinical implications.

Establishing the normal ranges for continuous variable biomarkers, such as physiological, laboratory, and imaging parameters, is essential for their clinical applications. A commonly employed method for this purpose involves measuring these biomarkers in a sample of healthy individuals and determining either the 5th and 95th or the 1st and 99th percentiles to demarcate the lower and upper limits of normal. In the case of strain, particularly, the LLN holds greater clinical significance as lower strain values are indicative of abnormality. This threshold is arguably more crucial than assessing the mean value. Additionally, considering the error and uncertainty associated with every calculated estimate, including LLN, it is critical to report the 95%CI around the LLN. Applying this method allows the LLN to be clinically interpreted in two ways. For example, in the case of RA strain measured by TomTec in this study, the LLN with its associated 95%CI for RASr is 29.6 (26.5, 32.7). This is traditionally interpreted as RASr higher than 29.6% is normal and lower than 29.6% is abnormal. Taking into account the 95%CI and errors with all biological measurements, however, means that RASr should be interpreted as values above 32.7% are normal, below 26.5% is abnormal and between 26.5–32.7% is borderline, as there is uncertainty around where the LLN is but most likely within that 95%CI [10, 11].

The current American Society of Echocardiography (ASE) guidelines dating back to 2015 suggested using 25% as the LLN for RA strain. However, this is not well established[1]. A prior subject-level meta-analysis reported that 15 studies in 2469 healthy subjects were selected for analysis. The normal range values for RA strain were reservoir 42.7% (95% CI, 39.4 to 45.9%), conduit 23.6% (95% CI, 20.7 to 26.6%), and contractile16.1% (95% CI, 13.6 to 18.6%) [9]. Another meta-analysis including 4,111 subjects from 21 studies showed that the pooled mean RA strains were reservoir 44% (95% CI 25%-63%), conduit 18% (95% CI 7%-28%), and contractile 17% (95% CI 2%-32%)[8]. For mean RASr, our TomTec and VVI values are similar to prior meta-analyses, while EchoPAC and Epislon are slightly lower in magnitude. A subsequent study from the World Alliance Societies of Echocardiography reported a mean RASr of 46 ± 12% (45 ± 12% in men and 48 ± 13% in women), a slightly higher magnitude than our findings[12]. Variations in reported means and LLN of RA strains across studies leading to significant heterogeneity seen in meta-analyses are likely associated with differences in subject demographics and echocardiography equipment and software. The observed differences in RA strain measurements among vendors are contributed by variations in proprietary speckle-tracking methodologies, software algorithms, and strain calculation definitions unique to each vendor. These also include differences in myocardial border detection, region of interest selection, frame rate optimization, how peak strain is defined and measured, and spatial–temporal resolution. Our findings further emphasize the need that until all techniques have standardized method of RA strain, the same software should be used for serial monitoring of RA strain, and caution and LLNs be accounted for when comparing strain measured on different strain vendor software. Prospectively establishing means and LLNs in local populations when newer strain measurement techniques and software are applied for wider clinical use should be considered rather than solely relying on thresholds reported in prior guidelines and studies.

In our regression analyses, we found that strain software and age were key parameters affecting two of three types of RA strain measurements each. It has been shown previously that as we age, the reservoir and conduit phase functions of both atria decrease in magnitude, which we also found, while the contractile phase function is more variable [13, 14]. Similarly, smaller studies have also shown age as seen in our results, blood pressure, and heart rate variability as influencers of RA deformation parameters [15, 16]. Aging is associated with atrial myopathy, characterized by structural and electrical remodeling, including interstitial fibrosis and altered ion channel expression, which can reduce atrial strain [17]. Additionally, aging is associated with regional conduction slowing and increased prevalence of low-voltage areas in the atria [18]. Hemodynamic changes in conditions like PH or left heart failure and physiological states like exercise may also impact right-sided mechanics, leading to altered strain, contractility, or abnormal flow. Associations between RA strain and various factors, including right-ventricular systolic function, coronary artery disease, PH, and response to cardiac resynchronization therapy, have been observed [19–22]. In PH, RA strain provides valuable prognostic information. Hasselberg et al. demonstrated that RA peak longitudinal strain was independently associated with survival in patients with precapillary PH, adding prognostic value beyond traditional measures like RA area and pressure [23]. Similarly, D’Alto et al. found that RA reservoir function, measured by strain rate, was an independent predictor of clinical worsening in idiopathic pulmonary arterial hypertension [24]. Further, in heart failure, RA strain also holds prognostic significance. Jain et al. reported that RA reservoir and conduit strain were independent predictors of all-cause mortality in patients with heart failure with preserved and reduced ejection fraction [25]. This suggests that RA strain can provide additional prognostic information beyond the conventional measures of right-ventricular function. Interestingly, we found VVI to have the highest magnitude of mean RASr and RASct across vendors, while the opposite has been observed in studies for left- and right-ventricular systolic strain [26–29]. On the other hand, EchoPAC had lower magnitude RASr and RASct measurements than TomTech. These findings highlight the importance of still using the same vendor software (and, in the case of EchoPAC, using the same GE scanner) in the serial monitoring or comparisons of RA strain measurements. Further, the ASE, along with the European Association of Echocardiography and the Canadian Society of Echocardiography, recommends that serial strain assessments be performed using the same software to minimize variability and ensure consistency in longitudinal patient monitoring [30]. This recommendation is crucial for clinical practice, as inconsistent strain measurements can lead to misinterpretation of cardiac function and potentially impact clinical decision-making. In addition, the use of separate reference ranges of RA strain by strain software and possibly age group are suggested, and these parameters should be taken into account when interpreting RA strain values.

This study has some limitations. It is an observational cohort study, which inherently carries biases, although it is prospective in design. The sample size of 100 participants is relatively modest, especially when stratified across different age groups, genders, and scanners for subgroup analyses; therefore, external validation in larger healthy cohorts is warranted. In addition, left atrial strain methods were employed for RA strain measurements in this study similar to prior studies because of lack of standardized RA strain-specific modules by the main strain vendors, and this may also introduce accuracy and variability in RA strain measurements. The cohort comprised healthy individuals (majority Caucasian) from a United States tertiary cardiology center, which might limit the generalizability of the results to other populations with different demographic profiles worldwide. Each strain software used in the study has its own limitations in terms of measurement accuracy, leading to significant intra- and inter-reader agreement reported. As this study was cross-sectional and focused on healthy subjects, we did not evaluate outcomes during follow-up and prognostic utility of RA strain, which warrants future research.

## Conclusion

In conclusion, TomTec, VVI and Epislon measurements of RA strain were feasible on both GE and Philips scans, while EchoPAC could only measure on GE scans. We determined means, LLNs, and their 95%CIs for RA strains to distinguish between normal, borderline, and abnormal RA strains for clinical and research applications, although significant intra- and inter-reader variability were limitations. We also found strain software (especially EchoPAC or VVI versus TomTec) to significantly affect RASr and RASct measurements, while age was significantly associated with RASr and RAScd measurements. Further studies are necessary to validate the normal ranges we reported and to evaluate the prognostic value of abnormal and borderline RA strains.

## Data Availability

All the data related to this study are available with the corresponding author upon reasonable request.
